# Effect of microfracture and autologous-conditioned plasma application in the focal full-thickness chondral defect of the knee: an experimental study on rabbits

**DOI:** 10.1186/s13018-015-0254-0

**Published:** 2015-07-16

**Authors:** Mustafa Karakaplan, Nurzat Elmalı, Efe Mirel, Nurhan Şahin, Emre Ergen, Candan Elmalı

**Affiliations:** Orthopaedics and Traumatology Department, Turgut Ozal Medical Center, Inonu University Medical School, Malatya, 44100 Turkey; Bezmialem Vakıf University, Orthopaedics and Traumatology Clinic, Istanbul, Turkey; Kelkit State Hospital, Orthopaedics and Traumatology Department, Gumushane, Turkey; Pathology Department, Turgut Ozal Medical Center, Inonu University Medical School, Inonu, Malatya Turkey; Süreyya Pasa Hospital Pathology Clinic, Istanbul, Turkey

**Keywords:** Microfracture, Cartilage, Platelet-rich plasma, Periosteum

## Abstract

**Purpose:**

The aim of the present study was to evaluate the effect of microfracture and intraarticular autologous conditioned plasma (ACP) injection on cartilage regeneration in a focal full-thickness chondral defect model created in the knee joint.

**Methods:**

Full-thickness chondral defects of 3 × 6 mm^2^ were surgically created in right medial femoral condyles (MFC) of New Zealand rabbits, and the rabbits were then divided into three groups according to treatment: Group 1 received only microfracture (mfx), Group 2 received mfx plus intraarticular ACP, and Group 3 received mfx; the defect was covered by the periosteum, and then, ACP was applied subperiosteally and intraarticularly. Twelve weeks after injection, the animals were sacrificed and the femoral condyles were evaluated macroscopically and histologically by hematoxylin-eosin staining. Then, histological sections were scored using the International Cartilage Repair Society (ICRS) visual histological scale.

**Results:**

Findings showed that in both mfx/ACP-treated groups, the defects were filled regularly and smoothly, the defects had a greater fill and good integration into the surrounding host tissue, and the repair matrix had more hyaline-like character. On the other hand, defects were filled with an irregular, fibrous cartilage in the mfx-treated group. Histological scores in Group 2 and Group 3 were better compared to Group 1.

**Conclusion:**

In the present study, we were able to demonstrate a beneficial effect of intraarticular administration of ACP as a coadjuvant of microfractures in order to regenerate hyaline-like cartilage in full-thickness chondral lesions in a rabbit model.

## Background

It has been well established that articular cartilage has poor intrinsic capacity for self-repair due to the low cellular mitotic activity of chondrocytes and its avascularity [1]. Although many treatment options are currently available, such as microfracture, osteochondral grafting, and autologous chondrocyte implants, none of these options fulfill the criteria for an ideal repair solution, including a hyaline repair tissue that completely fills the defect and integrates well with the surrounding normal cartilage [2, 3]. The microfracture technique is currently a common first-line treatment for patients with cartilage defects of the knee, resulting in the formation of a fibrocartilaginous repair tissue with inferior biomechanical properties compared to normal hyaline cartilage [4–6]. Therefore, research is continually being conducted in an attempt to find biological adjuvant treatments to improve the quality of the microfracture repair tissue, with the goal of producing a more hyaline-like repair, capable of durable, long-term functional improvement [7–13]. The original concept for clinical application of platelet-rich plasma (PRP) focused only on the concentration of platelets because platelets are a natural reservoir of many growth factors important for tissue healing. More recently, it is accepted that PRP, like all of the blood-derived biologics discussed herein, is a milieu of bioactive factors. Broadly, PRP preparations can be defined as buffy coat or plasma based. Autologous conditioned plasma (ACP) is a form of PRP that belongs in the plasma-based group. ACP, which is an autologous blood product produced by the centrifugation of whole blood, thereby yielding 2–8 times higher than baseline concentration of platelets, has produced various effects on sports injuries and cartilage [14–27]. The advantages of the use of ACP as an adjuvant to microfracture are the sealant properties for the implant, the capacity to promote cellular implantation, and the presence of growth factors that are involved in mesenchymal stem cells (MSC) chondrogenic differentiation [28].

The aim of the present study was to investigate the effect of intraarticular ACP injection, and intraarticular and subperiosteal ACP injections into the defect covered by periosteum on cartilage repair, and compare the results with the results of microfracture alone.

## Materials and methods

In this study, “Principles of laboratory animal care” (NIH publication No. 86–23, revised 1985) were followed. The research protocol was reviewed and approved by the Ethics Committee of the Experimental Animals of Faculty of Medicine in Inonu University. The study was carried out in the facilities of Orthopedics and Traumatology, Pathology and Experimental Animal Production and Research Center. Rabbits were obtained from Inonu University Experimental Animal Production and Research Center. Twenty-one mature (18 weeks old) New Zealand-type white rabbits, with a mean weight of 2450 g (1950–2900 g) were used in this study. Rabbits were divided into three groups of seven animals per group. One hour prior to the surgery, 75 mg/kg of cefazolin sodium (Sefazol®, Mustafa Nevzat, Turkey) was administered to all rabbits. All procedures were carried out under aseptic conditions, using intramuscular anesthesia with ketamine 35 mg/kg (Ketalar®, Pfizer, USA), xylazine 5 mg/kg (Rompun®, Bayer, Germany), and acepromazine 1 mg/kg (Plegicil®, Sanofi, Turkey), enrofloxacin 10 mg/kg (Baytril-K®, Bayer, Germany), and tramadol 4 mg/kg (Contramal®, Abdi Ibrahım, Turkey) were administered to all animals preoperatively and up to 2 days after surgery.

### Preparation of autologous-conditioned plasma

Prior to blood drawing, 1 ml of ACD-A (Anticoagulant Citrate Dextrose Solution; NoClot 400, Cytosol Laboratories, Inc.) was drawn into the outer syringe of the ACP Double syringe system (Arthrex Inc., FL 34108 USA). Then, 9 ml of venous blood was drawn from the posterior auricular vein, and blood samples were centrifuged at 1500 rpm for 5 min (Hettich Rotofix 32A). Approximately 2 ml of thrombocyte-rich plasma (ACP) obtained from erythrocytes and leukocytes were drawn into the inner syringe. According to the manufacturer’s reference, this procedure results in a 3-fold increase in thrombocyte count and a 2- to 6-fold increase in growth factor levels [25].

### Surgical procedure

The lower extremities of the animals were prepared appropriately and then covered. After crossing the cutaneous and subcutaneous fascia along the right knee midline using a longitudinal incision, the knee joint was approached by dislocating patella laterally with using parapatellar medial arthrotomy. A 3 × 6-mm-wide full-thickness chondral defect was created in the load-bearing region of the medial femoral condyle by using a hand perforator (Aesculap®) and a 2.7-mm-long drill cap according to the method described by Hui et al. [29]. After creation of the cartilage defect, each specimen then underwent microfracture using a 0.032-in (0.8 mm) fine wire. Animals were divided into three groups. In Group 1, microfracture holes penetrating the subchondral bone plate were created in the periphery of the defect first and then into the center of the defect as described previously, leaving 1- to 2-mm bone bridges between the holes. In Group 2, the osteochondral defect was created similarly to Group 1, and microfracture was applied. Then, 2 cc of ACP was injected intraarticularly. In Group 3, an osteochondral defect was created and microfracture applied before defects were closed by suturing a 5 × 5-mm periosteum obtained from the anteromedial tibia onto the defect. Fibrin glue (Beriplast-p®, FarmaTek, Turkey) was used to ensure that periosteum covered the defect tightly. Then, 2 cc of ACP was administered under the periosteum and intraarticularly. The patella was reduced in all groups after the completion of the procedures. The arthrotomies were then closed in layers with a 4.0 vicryl suture in the deep layer and 3.0 nylon to the skin. After surgery, the animals were kept in individual cages (40 cm × 40 cm × 60 cm) at constant temperature and humidity, with 12:12-h light–dark cycle and unrestricted access to a standard diet and water. Animals were allowed to walk freely with full weight bearing and without immobilization. For the first 24 h after surgery, 75 mg/kg of cefazolin sodium was administered intramuscularly in three doses.

No complications were observed. At 12 weeks post-intervention, the rabbits were killed using an intravenous overdose of pentobarbital and the right condyles were dissected and subjected to macroscopic and microscopic analyses. For histological analysis, condyles were fixed in formalin, decalcified in nitric acid, and embedded in paraffin. Ten-micrometer-thick sagittal cross sections were cut through the tissue, and sections were stained with hematoxylin and eosin. The samples were evaluated independently by two pathologists and graded according to the International Cartilage Repair Society (ICRS) histological assessment of cartilage repair [30]. The scale was comprised of six categories, which assigned scores to the most prominent feature on each sample. The highest score (3) was assigned to the ideal repair result (i.e., truly regenerated tissue), and the lowest score (0) was assigned to the poorest repair result (Table 1).

**Table 1:** International Cartilage Repair Society (ICRS) evaluation form

Feature	Score
I. Surface	
Smooth/continuous	3
Discontinuities/irregularities	0
II. Matrix	
Hyaline	3
Mixture: hyaline/fibrocartilage	2
Fibrocartilage	1
Fibrous tissue	0
III. Cell distribution	
Columnar	3
Mixed/columnar-clusters	2
Clusters	1
Individual cells/disorganized	0
IV. Cell population viability	
Predominantly viable	3
Partially viable	1
<10 % viable	0
V. Subchondral bone	
Normal	3
Increased remodeling	2
Bone necrosis/granulation tissue	1
Detached/fracture/callus at base	0
VI. Cartilage mineralization (calcified cartilage)	
Normal	3
Abnormal/inappropriate location	0

### Statistical analysis

SPSS for Windows version 13.0 and Medcalc version 12.3.0 statistical softwares were used to analyze the histopathological differences between the groups and for data evaluation. Descriptive criteria for quantitative variables were presented as median (min–max). Kruskal–Wallis variance analysis and Conover post hoc test were used to compare the parameters between the groups (Table 2). *P* < 0.05 was considered statistically significant.

**Table 2:** The results of the comparison with the Conover post hoc test in pairs

Groups	Surface	Matrix	Cell distribution	Cell viability	Subchondral bone	Calcified cartilage
Group 1–2	*P* < 0.05	*P* > 0.05	*P* < 0.05	*P* < 0.05	*P* < 0.05	*P* > 0.05
Group 1–3	*P* < 0.05	*P* < 0.05	*P* < 0.05	*P* < 0.05	*P* < 0.05	*P* < 0.05
Group 2–3	*P* < 0.05	*P* < 0.05	*P* > 0.05	*P* > 0.05	*P* < 0.05	*P* > 0.05

### Ethics

This study has been approved by the appropriate ethics committee and has therefore been performed in accordance with ethical standards laid down in the 1964 Declaration of Helsinki and its later amendments.

## Results

### Macroscopic findings

**Fig. 1:**
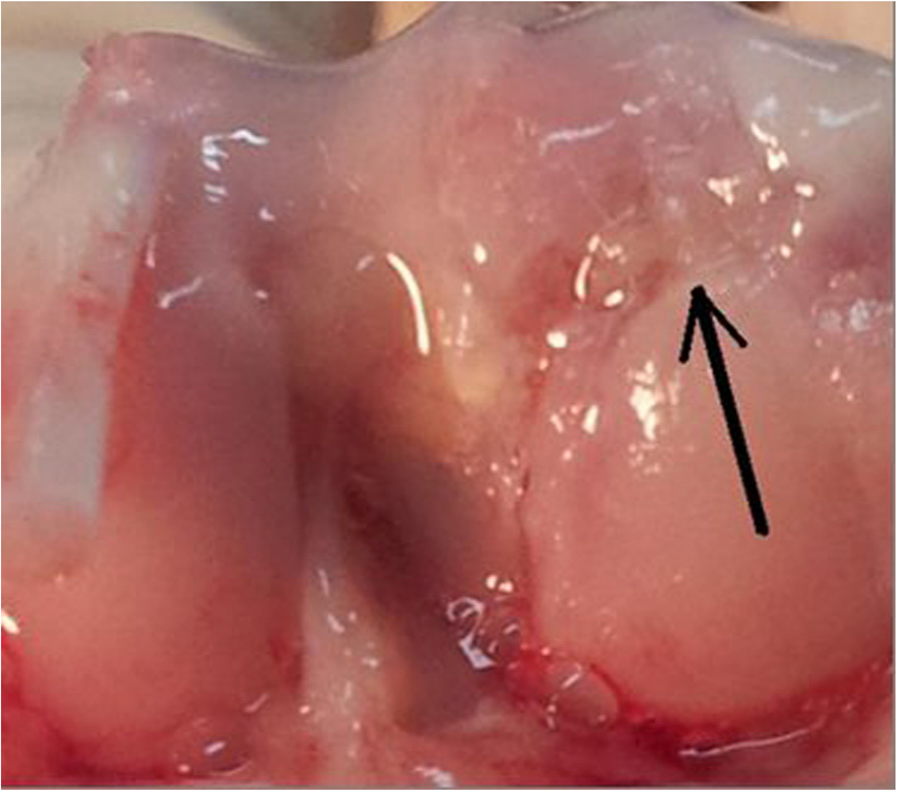
Group 1 (microfractures): surface of defect(arrow) showed surface irregularities

**Fig. 2:**
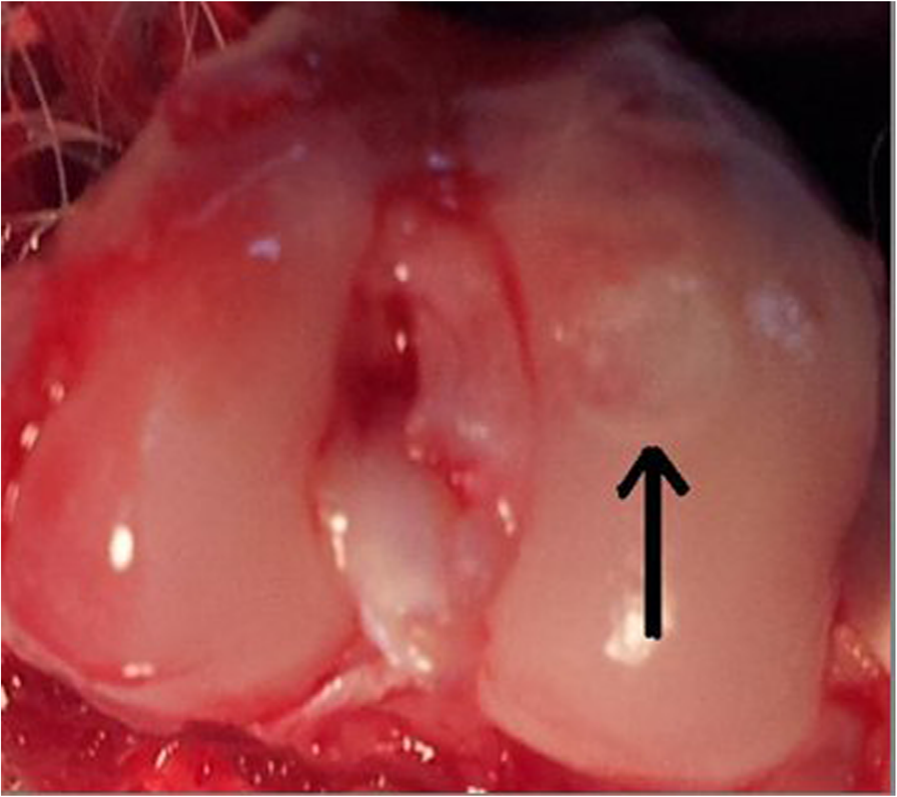
Group 2 (microfractures + ACP): repaired tissue (*arrow*) shows good integration with healthy surrounding cartilage

**Fig. 3:**
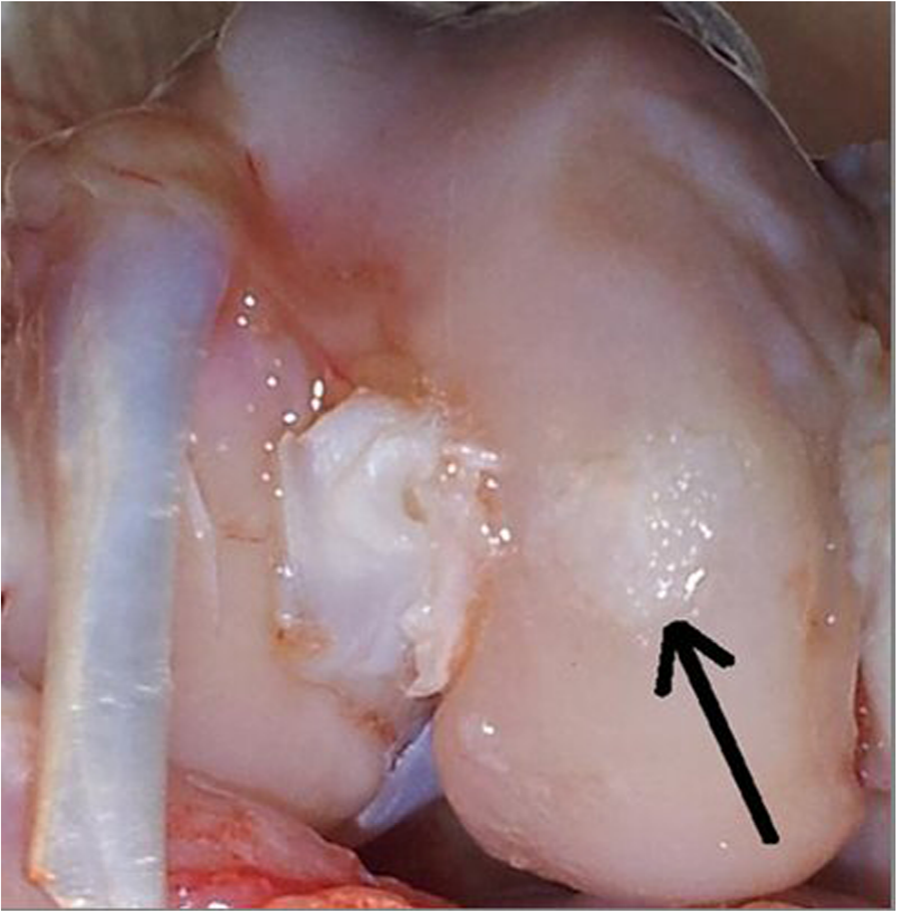
Group 3 (microfracture + periost + ACP): repaired tissue covers almost completely the defect(arrow), greater regularity, smooth surfaces, and a surface color similar to the surrounding cartilage

At the end of the study, macroscopic observation showed surface irregularities, with a purple-whitish appearance only in Group 1 (Fig. 1), which was treated with only microfracture (mfx). Macroscopic observation of the defect surface in ACP-treated Group 2 (Fig. 2) and Group 3 (Fig. 3) showed greater regularity, smooth surfaces, and a surface color similar to the surrounding cartilage.

### Histological findings

**Fig. 4:**
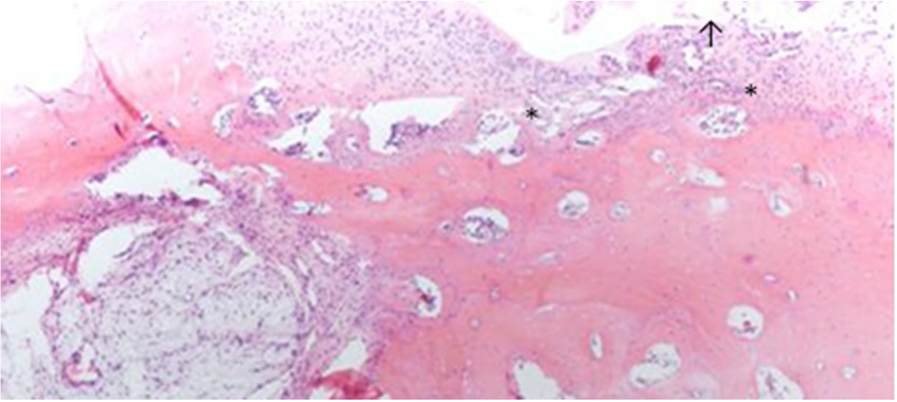
In the mfx-treated group (Group 1), all of the samples presented an irregular cartilage surface (*arrow*), a surface with a fibrocartilage matrix, and predominantly disorganized cell distribution (*asterisks*) (H&E)

**Fig. 5:**
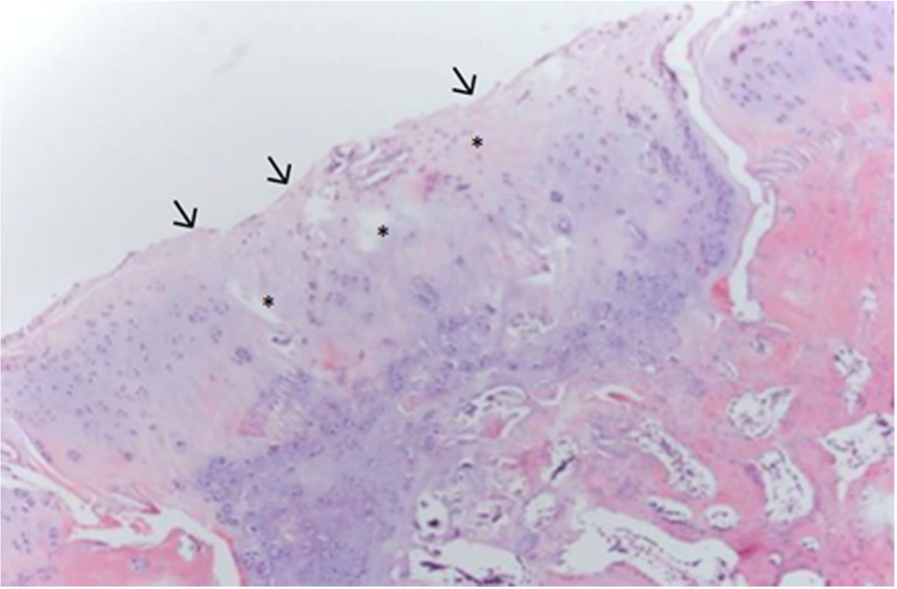
In the mfx plus intraarticular ACP treated group (Group 2), there are more regular surface (*arrows*), hyaline matrix mixed with fibrocartilage, in cell distribution criteria, disorganized cell distribution (*asterisks*) (H&E)

**Fig. 6:**
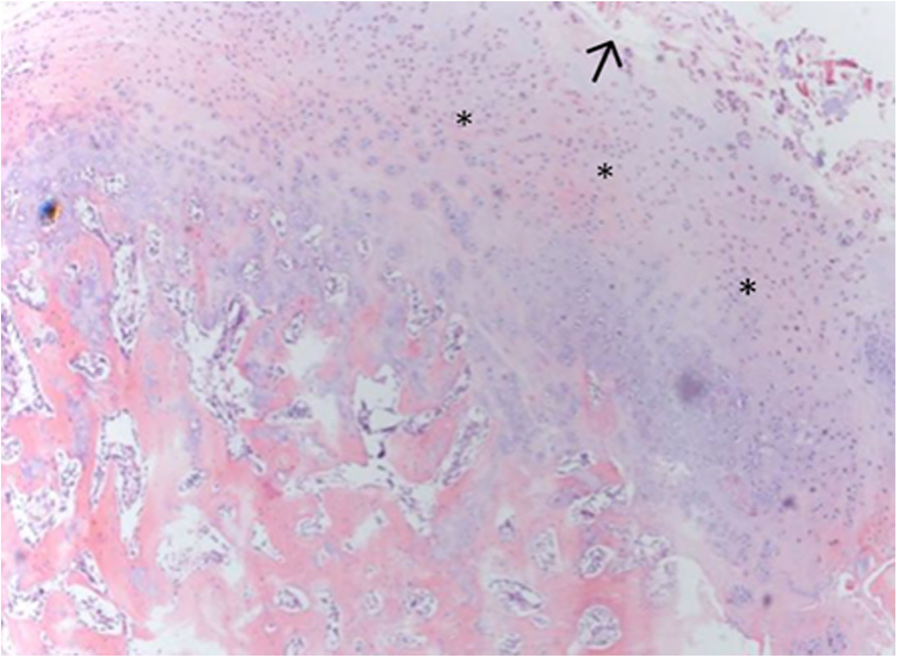
In the Group 3, after mfx, the defect was covered by the periosteum, and then, ACP was applied subperiosteally and intraarticularly. The tissue surface was mostly regular (*arrows*), with a hyaline matrix mixed with fibrocartilage in some areas but with chondrocytes more organized (*asterisks*) (H&E)

In the mfx-treated group (group 1), all of the samples presented an irregular cartilage surface, a surface with a fibrocartilage matrix, and predominantly disorganized cell distribution (Fig. 4). The mfx/ACP-treated group (Group 2) showed mainly a more regular surface, hyaline matrix mixed with fibrocartilage, and differences in cell distribution criteria, ranging from samples with a disorganized cell distribution to others with mixed columnar to cluster distribution (Fig. 5). In Group 3, the tissue surface was regular, with a hyaline matrix mixed with fibrocartilage in some areas but with chondrocytes more organized in the tissue compared to the mfx/ACP-treated group (Fig. 6). Cartilage mineralization was unaltered in all groups. When compared with both mfx/ACP-treated groups, the mfx group showed a significant reduction in scores in all categories (Table 3, *P* < 0.05). No difference was found in the interobserver histological grading.

**Table 3:** Mean ICRS scores of groups

Groups	Surface	Matrix	Cell distribution	Cell viability	Subchondral bone	Calcified cartilage
	Median (min–max)	Median (min–max)	Median (min–max)	Median (min–max)	Median (min–max)	Median (min–max)
Group 1 (*n* = 7)	0.0000 (0–0)	1.7143 (1–2)	1.8571 (1–2)	0.7143 (0–1)	1.4286 (1–2)	0.8571 (0–3)
Group 2 (*n* = 7)	1.7143 (0–3)	2.2857 (2–3)	2.5714 (2–3)	2.1429 (1–3)	2.2857 (2–3)	2.1429 (0–3)
Group 3 (*n* = 7)	3.0000 (3–3)	2.8571 (2–3)	2.8571 (2–3)	3.0000 (3–3)	3.0000 (3–3)	3.000 (3–3)
P (Kruskal Wallis)	0.0012	0.0034	0.0049	0.0010	0.0005	0.0207

## Discussion

In the present study, we found that ACP injection intraarticularly or both intraarticularly and subperiosteally into a defect covered by periosteum may enhance cartilage repair in the treatment with microfracture of focal full-thickness chondral defect in the knee.

Microfracture is the most well-studied marrow-stimulation procedure and involves the arthroscopic penetration of the subchondral plate with an awl. The creation of channels in the subchondral plate allows for the influx of blood, blood-derived cells, and bone marrow-derived mesenchymal stem and progenitor cells (MSCs) into the defect, forming a blood clot populated with platelets, growth factors, and bone marrow-derived pluripotent stem cells to allow for the remodeling of the fibrin clot in the defect into fibrocartilage repair tissue [4–9]. Although microfractures provide good short-term outcomes for many patients, mid- to long-term studies have demonstrated gradual decreases in functional outcomes after 24 to 36 months, potentially due to tissue degradation overtime [4, 5, 11, 13]. This could be explained because of the less durable fibrocartilage produced after microfracture and the poor integration of this tissue with the native cartilage. This fact is also consistent with the results of the current study, since in the mfx treated group, the newly formed tissue that filled the defect was fibrocartilage. Therefore, methods for the advancement of the microfracture technique are demanded that may enhance the content of key matrix components and improve the cartilaginous repair tissue such as scaffold enhancement, hyaluronic acid viscosupplementation, cytokine modulation techniques, and PRP injections [7–13].

The simple rationale supporting the use of PRP to treat cartilage injuries lies in the concept that PRP provides a milieu of bioactive growth factors such as transforming growth factor beta (TGF-β), platelet-derived growth factor (PDGF), insulin-like growth factor-1 (IGF-1), and vascular endothelial growth factor (VEGF) that have synergistic effects on cartilage matrix synthesis and mitigate the effects of catabolic cytokines such as interleukin-1 (IL-1) and tumor necrosis factor-α (TNF-α). PRP was also found to increase the cell viability of chondrocytes, the migration and chondrogenic differentiation of MSCs, increasing chondrocyte and mesenchymal stem cell proliferation, proteoglycan deposition, and type II collagen deposition. Different results have been reported on the application of PRP in chondral lesions. Not all studies concluded that PRP has a positive effect on cartilage repair [31]. In the study by Vaisman et al., the use of intraarticular BMS or PRP as coadjuvants to the microfracture technique for the treatment of acute chondral lesions was not associated with a significant improvement of hyaline cartilage regeneration [32]. This conclusion was only valid for the dosage used in that particular study (one intraarticular dose of 0.4 mg of betamethasone). Perhaps the PRP concentration was not sufficient to promote chondral healing. On the other hand, results from most animal studies have identified the potential utility of autologous platelets, both in isolation and as an adjunct to surgical procedures, to restore normal hyaline cartilage in articular injuries. The results of these studies have shown that the platelet supernatant stimulate chondrocyte proliferation and may form a scaffold for chondrocytes. Chondrocytes stimulated with PRP in vitro has been shown to increase synthesis of proteoglycans and collagen with the repair tissue generated after PRP treatment, demonstrating similar histological and biomechanical characteristics to normal hyaline cartilage [15–28, 32]. Milano et al. performed experimental studies on the effect of autologous PRP with microfracture on chondral defects in a sheep model and reported that treatment with PRP revealed an improvement of cartilage stiffness and showed higher ICRS scores [22, 23]. Similar positive results in chondral defect reconstruction with autologous platelets were found in the present study. An improvement in macroscopic observation was observed in groups that were treated with ACP, and histological analysis further supported these findings. In fact, within the criteria evaluated according to the histological ICRS score, all criteria showed a significant improvement with the use of ACP associated with microfracture compared to the microfracture treatment alone. ACP injection might effectively modify the joint microenvironment in order to facilitate cartilage regeneration. In the present study, in addition to the intraarticular ACP application, we also investigated the effectiveness of both ACP application into the defect covered by the periosteum and intraarticular ACP application. In Groups 2 and 3, hyaline-like cartilage was observed in knee defects; however, hyaline-like cartilage was more abundant in Group 3. In the same groups, all animals presented better chondral cellularity and regeneration and lower fibrosis. Among all groups, we observed the poorest healing in Group 1 and the best healing in Group 3. Furthermore, we discovered that augmenting the defect using periosteum to cover the defect (as a scaffold) and subperiosteal and intraarticular ACP injection was superior over microfracture application with respect to cartilage repair.

We believe that periosteum both contributes to joint regeneration, and as a scaffold, it can maintain thrombocyte concentration and growth factors for a longer time within ACP, thus, providing an advantage in obtaining a more hyaline-like cartilage and better results in periosteum-administered groups. Moreover, we believe that reporting different results after PRP application results from the differences in the methods of obtaining PRP, its concentration, method of activation, dose, and method for applying PRP [1]. In the current study, the preparation of PRP was performed according to the protocol described by Borzini et al. [15]. In their study, autologous PRP was used without performing a platelet count, which constitutes evidence that PRP can be prepared using this protocol.

This is a preliminary study, and hence, has certain limitations. The current study included a histological evaluation of the repair tissue for only up to 12 weeks after surgery and on a limited number of animals as well as periosteum that have many disadvantages that might not have been obvious in this short-term follow-up. Increasing the number of animals, and creating groups to include longer and different time periods, would increase the strength of the study. We only evaluated morphological parameters and could not demonstrate the effectiveness of ACP at the molecular levels. The lack of mechanical evaluation of the repair tissue is a shortcoming of this study. We did not analyze the plasma thrombocyte levels in the present study. According to the manufacturer’s reference, this procedure results in a 3-fold increase in thrombocyte count and a 2- to 6-fold increase in growth factor levels (EGF, VEGF, TGF-β, PDGF) [14, 28].

In conclusion, in the present study, we were able to demonstrate a beneficial effect of intraarticular administration of platelet-rich plasma as a coadjuvant of microfractures in order to regenerate hyaline-like cartilage in full-thickness chondral lesions in a rabbit model. Further researches with well-designed randomized controlled trials (RCTs) are required to validate the findings of this study in clinical settings.

## References

[1] Buckwalter JA (1999). Evaluating methods for restoring cartilaginous articular surfaces. Clin Orthop Relat Res.

[2] Lim HC, Bae JH, Song SH, Park YE, Kim SJ (2012). Current treatments of isolated articular cartilage lesions of the knee achieve similar outcomes. Clin Orthop Relat Res.

[3] van Osch GJ, Brittberg M, Dennis JE, Bastiaansen-Jenniskens YM, Erben RG, Konttinen YT, Luyten FP (2009). Cartilage repair: past and future—lessons for regenerative medicine. J Cell Mol Med.

[4] Mithoefer K, Williams RJ, Warren RF, Potter HG, Spock CR, Jones EC, Wickiewicz TL, Marx RG (2006). Chondral resurfacing of articular cartilage defects in the knee with the microfracture technique. Surgical technique. J Bone Joint Surg Am.

[5] Steadman JR, Briggs KK, Rodrigo JJ, Kocher MS, Gill TJ, Rodkey WG (2003). Outcomes of microfracture for traumatic chondral defects of the knee: average 11-year follow-up. Arthroscopy.

[6] Gill TJ, Asnis PD, Berkson EM (2006). The treatment of articular cartilage defects using the microfracture technique. J Orthop Sports Phys Ther.

[7] Erggelet C, Neumann K, Endres M, Haberstroh K, Sittinger M, Kaps C (2007). Regeneration of ovine articular cartilage defects by cell-free polymer-based implants. Biomaterials.

[8] Strauss EJ, Barker JU, Kercher JS, Cole BJ, Mithoefer K (2010). Augmentation strategies following the microfracture technique for repair of focal chondral defects. Cartilage.

[9] Frisbie DD, Oxford JT, Southwood L, Trotter GW, Rodkey WG, Steadman JR, Goodnight JL, McIlwraith CW (2003). Early events in cartilage repair after subchondral bone microfracture. Clin Orthop Relat Res.

[10] Fortier LA, Barker JU, Strauss EJ, McCarrel TM, Cole BJ (2011). The role of growth factors in cartilage repair. Clin Orthop Relat Res.

[11] Gudas R, Kalesinskas RJ, Kimtys V, Stankevicius E, Toliusis V, Bernotavicius G, Smailys A (2005). A prospective randomized clinical study of mosaic osteochondral autologous transplant versus microfracture for the treatment of osteochondral defects in the knee joint in young athletes. Arthroscopy.

[12] Gunes T, Bostan B, Erdem M, Koseoglu RD, Asci M, Sen C (2012). Intraarticular hyaluronic acid injection after microfracture technique for the management of full-thickness cartilage defects does not improve the quality of repair tissue. Cartilage.

[13] Knutsen G, Engebretsen L, Ludvigsen TC, Drogset JO, Grontvedt T, Solheim E, Strand T, Roberts S, Isaksen V, Johansen O (2004). Autologous chondrocyte implantation compared with microfracture in the knee: a randomized trial. J Bone Joint Surg Am.

[14] Garcia-Alvarez F, Castiella T, Grasa JM, Monzón M, Laclériga A, Palanca D (2005). Autologous platelets and articular surface repair in an experimental model. J Orthop Sci.

[15] Borzini P, Mazzucco L (2005). Tissue regeneration and in loco administration of platelet derivatives: clinical outcome, heterogeneous products, and heterogeneity of the effector mechanisms. Transfusion.

[16] Eppley BL, Woodell JE, Higgins J (2004). Platelet quantification and growth factor analysis from platelet-rich plasma: implications for wound healing. Plast Reconstr Surg.

[17] Hall MP, Band PA, Meislin RJ, Jazrawi LM, Cardone DA (2009). Platelet-rich plasma: current concepts and application in sports medicine. J Am Acad Orthop Surg.

[18] Kaps C, Loch A, Haisch A, Smolian H, Burmester GR, Haupl T, Sittinger M (2002). Human platelet supernatant promotes proliferation but not differentiation of articular chondrocytes. Med Biol Eng Comput.

[19] Krüger JP, Hondke S, Endres M, Pruss A, Siclari A, Kaps C (2012). Human platelet-rich plasma stimulates migration and chondrogenic differentiation of human subchondral progenitor cells. J Orthop Res.

[20] Ksander GA, Sawamura SJ, Ogawa Y, Sundsmo J, McPherson JM (1990). The effect of platelet releasate on wound healing in animal models. J Am Acad Dermatol.

[21] McCarrel T, Fortier L (2009). Temporal growth factor release from platelet rich plasma, trehalose lyophilized platelets, and bone marrow aspirate and their effect on tendon and ligament gene expression. J Orthop Res.

[22] Milano G, Deriu L, Sanna Passino E, Masala G, Manunta A, Postacchini R, Saccomanno MF, Fabbriciani C (2012). Repeated platelet concentrate injections enhance reparative response of microfractures in the treatment of chondral defects of the knee: an experimental study in an animal model. Arthroscopy.

[23] Milano G, Sanna Passino E, Deriu L, Careddu G, Manunta L, Manunta A, Saccomanno MF, Fabbriciani C (2010). The effect of platelet rich plasma combined with microfractures on the treatment of chondral defects: an experimental study in a sheep model. Osteoarthritis Cartilage.

[24] Woodell May J, Matuska A, Oyster M, Welch Z, O’Shaugh Nessey K, Hoeppner J (2011). Autologous protein solution inhibits MMP-13 production by IL-1beta and TNF alpha-stimulated human articular chondrocytes. J Orthop Res.

[25] Hapa O, Çakici H, Yüksel HY, Fırat T, Kükner A, Aygün H (2013). Does platelet-rich plasma enhance microfracture treatment for chronic focal chondral defects? An in-vivo study performed in a rat model. Acta Orthop Traumatol Turc.

[26] Sun Y, Feng Y, Zhang CQ, Chen SB, Cheng XG (2010). The regenerative effect of platelet-rich plasma on healing in large osteochondral defects. Int Orthop.

[27] Taylor DW, Petrera M, Hendry M, Theodoropoulos JS (2011). A systematic review of the use of platelet-rich plasma in sports medicine as a new treatment for tendon and ligament injuries. Clin J Sport Med.

[28] Sanchez M, Anitua E, Orive G, Mujika I, Andia I (2009). Platelet-rich therapies in the treatment of orthopaedic sport injuries. Sports Med.

[29] Hui JH, Chen F, Thambyah A, Lee EH (2004). Treatment of chondral lesions in advanced osteochondritis dissecans: a comparative study of the efficacy of chondrocytes, mesenchymal stem cells, periosteal graft, and mosaicplasty (osteochondral autograft) in animal models. J Pediatr Orthop.

[30] Varlet PM, Aigner T, Brittberg M, Ballough P, Hollander A, Hunziker E, Kandel R, Nehrer S, Pritzker K, Roberts S, Stauffer E (2003). Histological assessment of cartilage repair: a report by the histology endpoint committee of the international cartilage repair society (ICRS). J Bone Joint Surg.

[31] Smyth NA, Murawski CD, Fortier LA, Cole BJ, Kennedy JG (2013). Platelet-rich plasma in the pathologic processes of cartilage: review of basic science evidence. Arthroscopy Aug.

[32] Vaisman A, Figueroa D, Calvo R, Espinosa M, Melean P, Gallegosa M, Conget P (2012). Steroids and platelet-rich plasma as coadjuvants to microfracture for the treatment of chondral lesions in an animal model: can the healing be enhanced?. Cartilage.

